# Data in support of effect of blue LED irradiation in human lymphoma cells

**DOI:** 10.1016/j.dib.2016.01.018

**Published:** 2016-01-15

**Authors:** Phil-Sun Oh, Hyosook Hwang, Hwan-Seok Jeong, Jeongil Kwon, Hyun-Soo Kim, Minjoo Kim, SeokTae Lim, Myung-Hee Sohn, Hwan-Jeong Jeong

**Affiliations:** Department of Nuclear Medicine, Molecular Imaging & Therapeutic Medicine Research Center, Cyclotron Research Center, Institute for Medical Sciences, Biomedical Research Institute, Chonbuk National University Medical School and Hospital, Jeonju, Jeonbuk 561-712, Republic of Korea

## Abstract

As a new and preferred light source for phototherapy, blue light emitting diodes (LEDs) with wavelengths of 400–500 nm have been used to treat hyperbilirubinaemia in infantile jaundice [1]. Recent studies report that blue LED irradiation induces apoptosis by stimulating a mitochondrial pathway and reduces the early growth rate of melanoma cells in mice [2]. Here, we detected the induction of apoptotic cell death and formation of autophagosome in human B lymphoma cells after irradiation with blue LED. This paper provides data in support of the research article entitled “Blue light emitting diode induces apoptosis in lymphoid cells by stimulating autophagy” [3].

**Specifications table**

**Table:** 

Subject area	*Biology*
More specific subject area	*Apoptosis*
Type of data	*Image (confocal microscopy), graph*
How data was acquired	*Microscope, flow cytometry*
Data format	*Raw data, analyzed*
Experimental factors	*Blue LED irradiation*
Experimental features	*Apoptosis of blue LED-irradiated cells was detected by TUNEL staining for measuring DNA fragments and annexin V/PI staining to distinguish apoptotic and necrotic cells*
Data source location	*Jeonju, Republic of Korea*
Data accessibility	*Data are provided in this article*

**Value of the data**

• The data provide information about the effect of blue LED irradiation in B lymphoma cells.
• The data can be used to identify the interaction between autophagy and apoptosis.
• The data inform future study for application of the blue LED to kill cancer cells including lymphoma.

## 1 Data

*Here, we evaluated the increase of fragmented DNA, levels of intracellular superoxide anion* $\left(O_{2}^{•-}\right)$*, LC*3 *conversion and caspase activation in RAMOS cells. Irradiation with blue LED induced apoptotic cell death through autophagosome activation in RAMOS cells.*

## 2 Experimental design, materials and methods

### 2.1 Induction of apoptotic cell death under blue LED irradiation in RAMOS cells

**Fig. 1:**
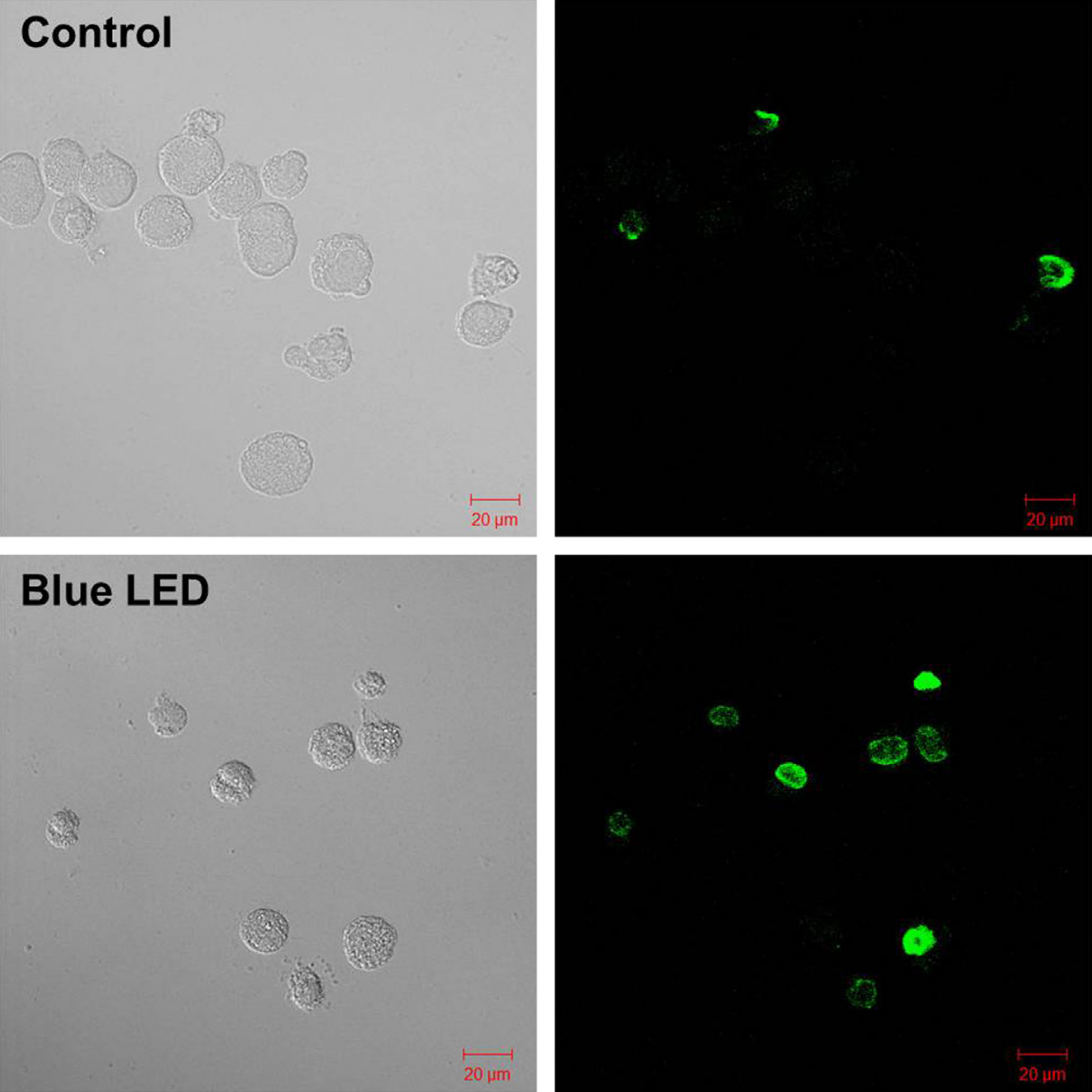
TUNEL staining of RAMOS cells. Cells were treated with the TdT enzyme and stained with dUTP-fluorescein isothiocyanate using a TUNEL staining kit (Takara Inc., Japan).

The human B cell lymphoma RAMOS cell line was grown in IMDM supplemented with 10% FBS, 10 μg/ml gentamicin, and 0.25 μg/ml amphotericin B. We used a blue LED with a wavelength of 450 nm at a power of 6.3 mW/cm^2^ for the experiment. RAMOS cells (1×10^6^ cells/well) were exposed to blue LED for 4 h. TUNEL staining to detect DNA fragmentation carried out according to the manufacturer׳s instructions (in situ Apoptosis Detection Kit, Takara, Japan). As shown in Fig. 1, numerous cells were stained green, indicating that apoptotic cell death occurred in RAMOS cells exposed to blue LED. The experiments were performed three times with similar results.

### 2.2 Autophagosome activation by blue LED irradiation in RAMOS cells

**Fig. 2:**
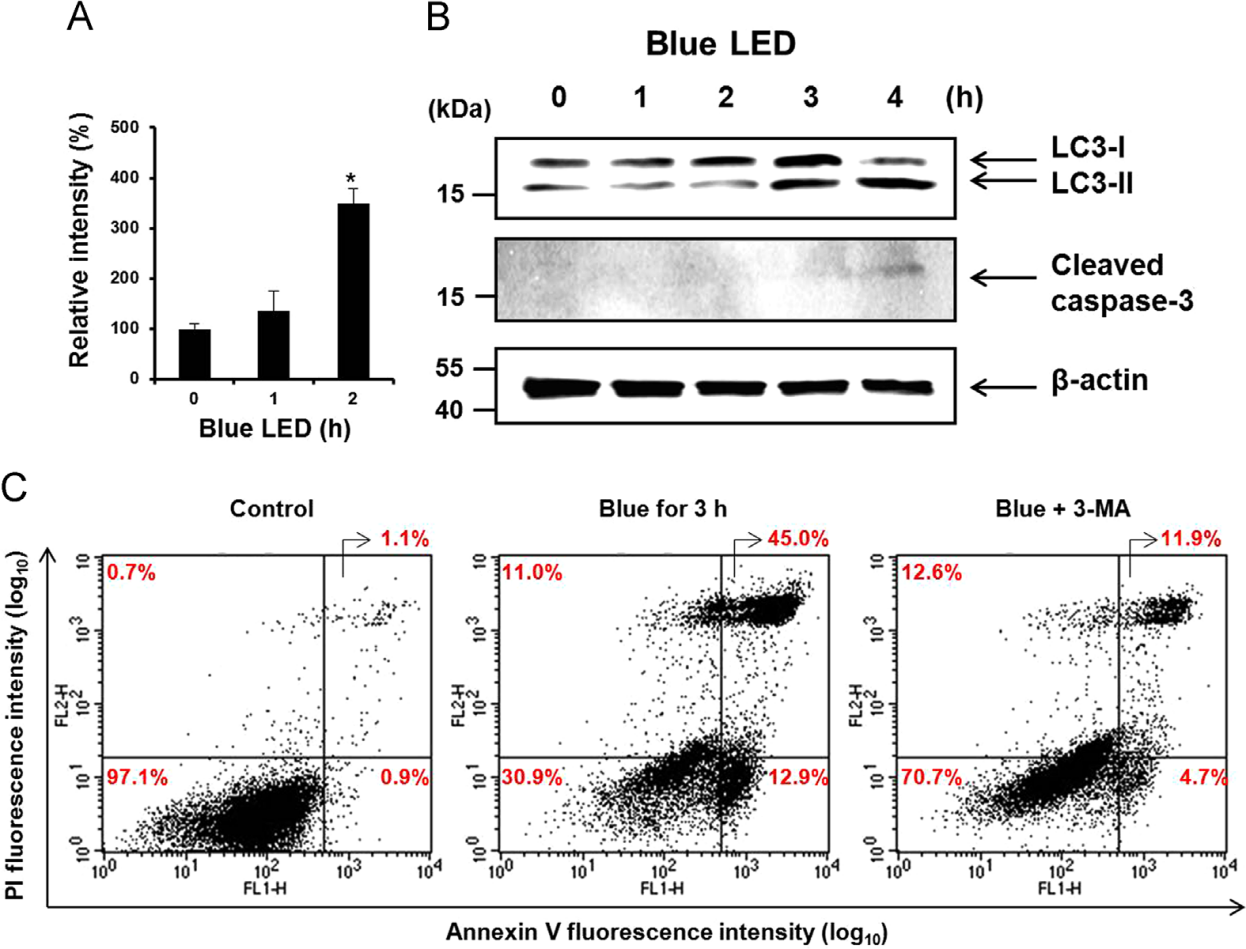
Apoptotic effect of blue LED via autophagy in RAMOS cells. (A) Intracellular O_2_^•-^ production using DHE staining was analyzed by flow cytometry. (B) Expression of caspase-3 and LC3-I/II under LED irradiation was determined via Western blot analysis. (C) The cells were exposed to blue LED in the presence of 3-MA for 3 h. FACS analysis indicated that cells with apparent early apoptosis (lower right quadrant) and late apoptosis (upper right quadrant) show positive annexin V-FITC staining. *p<0.05 compared with the control group.

Intracellular $\left(O_{2}^{•-}\right)$ levels were measured using an oxidation-sensitive fluorescent probe dye, dihydroethidium (DHE, Ex/Em=518 nm/605 nm), as previously described [3]. The $\left(O_{2}^{•-}\right)$ levels were increased up to 349.2% in blue LED-irradiated RAMOS cells at 2 h as compared to the control. Also, the band of cleaved caspase 3 showed weakly after 4 h exposure to blue LED (Fig. 2A). Protein preparation and western blotting were carried out as previously described [2], [3]. All experiments were performed at least three times and the results of the immunoblot assays were calculated as relative intensity using Image J software. LC3 conversion levels (LC3-I to LC3-II) were detected. LC3-II level which is positively correlated with the number of autophagosomes [4] increased by 139.3% and 221.3% after 3 h and 4 h of exposure to blue LED (Fig. 2B). To assess interaction between autophagy and apoptosis, 3-methyladenine (3-MA) which blocks the initiation stage of autophagy was pretreated. The cells were suspended in 500 μl annexin V working solution containing 5 μl of annexin V-FITC and 10 μl of propidium iodide (PI) for 15 min, according to the manufacturer׳s instructions. Fluorescence was measured by flow cytometer (Becton Dickinson, San Jose, CA) and analyzed with Cell Quest software (Becton Dickinson). Moreover, the population of annexin V positive cells was decreased in 3-MA (autophagy inhibitor)-pretreated RAMOS cells as compared to the blue LED group. After blue LED irradiation, the viable cells were 70.7%, the primary necrotic cells were 12.6%, the early apoptotic cells were 4.7%, and the late apoptotic/secondary necrotic cells were 11.9% in 3-MA-pretreated RAMOS cells, respectively.

## 3 Statistical analysis

All data represent the mean of at least three independent experiments. The results were analyzed using the unpaired two-tailed Student׳s *t*-test. *P*<0.05 was designated as the level of significance.
