# Supplementary material for: Data in support of effect of blue LED irradiation in human lymphoma cells
Source: Data Brief. 2016 Jan 15;6:630–3. doi: 10.1016/j.dib.2016.01.018 (PMC4735470; doi:10.1016/j.dib.2016.01.018)
Supplement: Supplementary file 1 — Supplementary material [file mmc1.doc]

**Conflicts of interest**

The authors have no financial conflicts of interest.
